# Maintenance of Board Certification in Ophthalmology; Who Cares?

**Published:** 2016

**Authors:** Fatemeh HEIDARY, Reza GHAREBAGHI

**Affiliations:** 1 Immunoregulation Research Center, Shahed University, Tehran, Iran; 2 International Virtual Ophthalmic Research Center (IVORC)

**Keywords:** Maintenance of Board, Exam, Medical Education, International Council of Ophthalmology (ICO), Certification, Educational Measurement, Ophthalmology

The issue of continuing education is vital for both physicians and patient safety, as it can protect physicians from the legal consequences of out-of-date management practices and protect patients from complications from out-of-date approaches. Following the pioneering establishment of the American Board of Ophthalmology in 1917, other organizations have established specialty board exams (1). The American Board of Medical Specialties (ABMS) established a time limit for certifications in the 1970s and now requires recertification. Specifically, physicians have to take a written examination every 6 to 10 years to be recertified (1-2). Physicians in Canada and the United Kingdom must undergo a similar process to maintain their certifications (3). 

Considering the above-mentioned policies, managers and organizations in charge of specialist training should definitely establish standards for satisfactory training programs for new graduates as well as board-certified specialists, especially university lecturers and trainers. These standards assure outstanding training in both practical and theoretical aspects of ophthalmology. Once these standards are established and their requirements are determined, the next step is to audit and evaluate the programs individually and to identify disadvantages that may be solved by implementing effective plans. 

In the past few decades, scientific developments in the field of ophthalmology have led to noticeable changes in both medical and surgical management. Targeted therapies have replaced traditional medical treatments, and there has been a significant movement toward the global implementation of diagnostic procedures. In view of these advances, continuing education is crucial to keeping abreast of the scientific changes in this rapidly developing field. In addition, international prerequisites for quality of healthcare services and knowledge motivate nations to adopt global assessment systems for healthcare professionals. 

One well-known organization upholding such systems is the International Council of Ophthalmology (ICO), which was established to encourage education in ophthalmology by “teaching the teachers”. The mission of the ICO is to develop efficient teaching programs and materials and to subsequently improve ophthalmic care worldwide. To achieve this goal, the ICO has asked lecturers and the heads of ophthalmologic societies to redefine and rethink ophthalmology training (4). 

The ICO has established independent exams that are free of external influence and can be conducted in the candidate’s home country. These standard examinations cover subject areas, such as basic science, optics and refraction, use of instruments, and clinical sciences. Once a candidate passes all three steps of these standard examinations or a comparable examination, the ICO will offer them an opportunity to take an advanced examination. If the candidate passes a local face-to-face examination in addition to the ICO advanced examination, he or she can use the post-nominal acronym FICO (Fellow of the ICO) (5).

Despite their high standards and excellent qualities, these exams are generally administered by ophthalmology trainees and residents. Instead, in most countries, board-certified ophthalmologists engage in training programs and education of residents in medical universities based on their interests. Currently, there is no standard examination for the re-evaluation and recertification of these ophthalmologists to ensure their competency in training duties and to ensure that their teaching skills are up-to-date. It may thus be useful for the ICO to institute another exam specifically for university lecturers, such as the MOCO (Maintenance of Certificate in Ophthalmology) exam. This exam would be in addition to other established and standardized ICO examinations for ophthalmology trainees and residents. Considering previous successes in using ICO examinations, the establishment of this new examination may improve ophthalmic education quality in all communities worldwide and thus eliminate the inequality gaps that exist within and between ICO member countries. This would improve standards of professionalism among trainees at university hospitals and may subsequently lead to improvements in the quality of care and education standards in all communities.

We hope that standard and fair international evaluations in ophthalmology and vision sciences may lead to the development of a more comprehensive and coherent international environment. Our proposal would drive the establishment of a more efficient universal alliance among ophthalmology societies and lead to improved patient safety and more standardized ophthalmology training.
